# Tri-element nanozyme PtCuSe as an ingenious cascade catalytic machine for the amelioration of Parkinson’s disease-like symptoms

**DOI:** 10.3389/fbioe.2023.1208693

**Published:** 2023-05-30

**Authors:** Hongdang Xu, Xin Ding, Lingrui Li, Qing Li, Zhiye Li, Hongqi Lin

**Affiliations:** ^1^ Department of Anesthesiology, Henan Provincial People^’^s Hospital, People^’^s Hospital of Henan University, Central China Fuwai Hospital, Central China Fuwai Hospital of Zhengzhou University, Zhengzhou, Henan, China; ^2^ Department of Anesthesiology, Pain and Perioperative Medicine, The First Affiliated Hospital of Zhengzhou University, Zhengzhou, Henan, China; ^3^ The Application Center for Precision Medicine, The Second Affiliated Hospital of Zhengzhou University, Zhengzhou, Henan, China

**Keywords:** Parkinson’s disease, nanozyme, PtCuSe, nerve cell damage, reactive oxygen species

## Abstract

Parkinson’s disease (PD), as the second most common neurodegenerative disease after Alzheimer’s, has become intractable with the increasing aging global population. The exploration of nanomedicine has broadened the opportunities for developing novel neuroprotective therapies. In particular, polymetallic functional nanomaterials have been widely used in the biomedicine field in recent years, exhibiting flexible and diversified functions and controllable properties. In this study, a tri-element nanozyme (PtCuSe nanozyme) has been developed with desirable CAT- and SOD-like activities for the cascade scavenging of reactive oxygen species (ROS). In particular, the nanozyme is suitable for relieving nerve cell damage by removing reactive oxygen species in cells and mitigating the behavioral and pathological symptoms in animal models of Parkinson’s disease. Therefore, this ingenious tri-element nanozyme may have potential in the treatment of Parkinson’s disease and other neurodegenerative diseases.

## 1 Introduction

Reactive oxygen species (ROS), which are generated in the oxygen metabolism process, contain various species, including hydroxyl radical (OH^.^), monomorphic oxygen (^1^O_2_), hydrogen peroxide radical (LOO^**.**^), hydrogen peroxide lipid (LOOH), nitroperoxyl (ONOO^−^), hypochlorous acid (HOCl), and ozone (O_3_), (Han et al., 2022; Zhang et al., 2022). Although a moderate amount of ROS can promote cell growth and energy metabolisms, excessive ROS would damage cell structures like mitochondria and DNA, causing cell death or apoptosis. It is generally believed that the pathophysiological processes of many neurodegenerative diseases, such as Parkinson’s disease, Alzheimer’s disease, and amyotrophic lateral sclerosis, are also closely related with ROS (Li et al., 2022a; Emin et al., 2022).

Parkinson’s disease (PD), as one of the most common neurodegenerative diseases worldwide, affects approximately 2% of people over 60 years of age (He et al., 2022a; Yuan et al., 2022). It is characterized by a wide spectrum of motor and non-motor symptoms, including resting tremors, bradykinesia, rigidity, cognitive impairments, and sleep disorders (Dilliard and Siegwart, 2023). It is believed that PD is caused by genetic and environmental factors, and the major neuropathological hallmark of PD is dopaminergic neuronal loss in the substantia nigra pars compacta (SNpc) (Wu et al., 2021; Cheng et al., 2022). However, recent studies have found that the occurrence and development of PD are closely related to oxidative stress and free radical generation. Patients with PD have high dopamine oxidation during metabolism to produce a large number of ROS, such as H_2_O_2_ and ultra-oxygen anion in the substantia nigra Fe^2+^catalytic, to further generate hydroxyl free radicals with higher toxicity (Xiong et al., 2020; Olson et al., 2021). Therefore, solving the problem of accumulation of excess free radicals to reduce the intracellular ROS level and alleviate neuronal degeneration damage is expected to be an effective strategy for treating the symptoms and root causes of PD based on an antioxidant system (Bengoa-Vergniory et al., 2020).

As a promising natural enzyme substitute, nanozymes possess both enzymatic activities and the characteristics of nanomaterials (Li et al., 2021; He et al., 2022b). PtCu bimetallic nanoalloys (PtCu NAs) are a new kind of bimetallic alloy nanozyme. Compared with traditional metal nanozymes, the noble metal has stable surface properties and adjustable size, which is supposed to artificially control the active structure of the nanozymes, giving them higher biocompatibility and cell uptake rate. PtCu NAs have been proven to have a variety of enzymatic activities, including superoxide dismutase, and the ability to remove intracellular reactive oxygen species and reduce intracellular oxidative stress (Li et al., 2022b; Gao et al., 2023; Yang et al., 2023). Previous studies have shown that PtCu NAs play a decisive role in blocking the prion-like spreading of nerve cells, and this mechanism has been reported in Alzheimer’s disease studies (Liu et al., 2021; Chen et al., 2022; Zhu et al., 2022). Obviously, in the future, PtCu NAs will play an irreplaceable role in more fields. Selenium is a constituent of glutathione peroxidase (GSH-Px) (Niu et al., 2021). Every mole of GSH-PX contains 4 g of selenium. Selenium is an important cofactor in GSH-PX and plays an unmatched role in catalyzing the redox reaction of reducing glutathione (GSH) with peroxide (Dringen et al., 2015; Peter et al., 2015; Sun et al., 2016). Therefore, it is an important cellular free radical scavenger in introducing selenium into PtCu nanozymes, which can improve biocompatibility and reduce biological toxicity (Huang et al., 2017; Hu et al., 2022). Therefore, PtCuSe shows great potential in catalyzing the generation of oxygen from over-produced hydrogen peroxide in cells, which reduces the damage caused by hydrogen peroxide to tissues and cells, solves the problem of accumulation of excess free radicals in order to reduce the intracellular ROS level and alleviate neuronal degeneration damage, and solves the problem of apoptosis in neurons in PD to a large extent (Ding et al., 2021; Xue et al., 2022; Yu et al., 2022).

**SCHEME 1:**
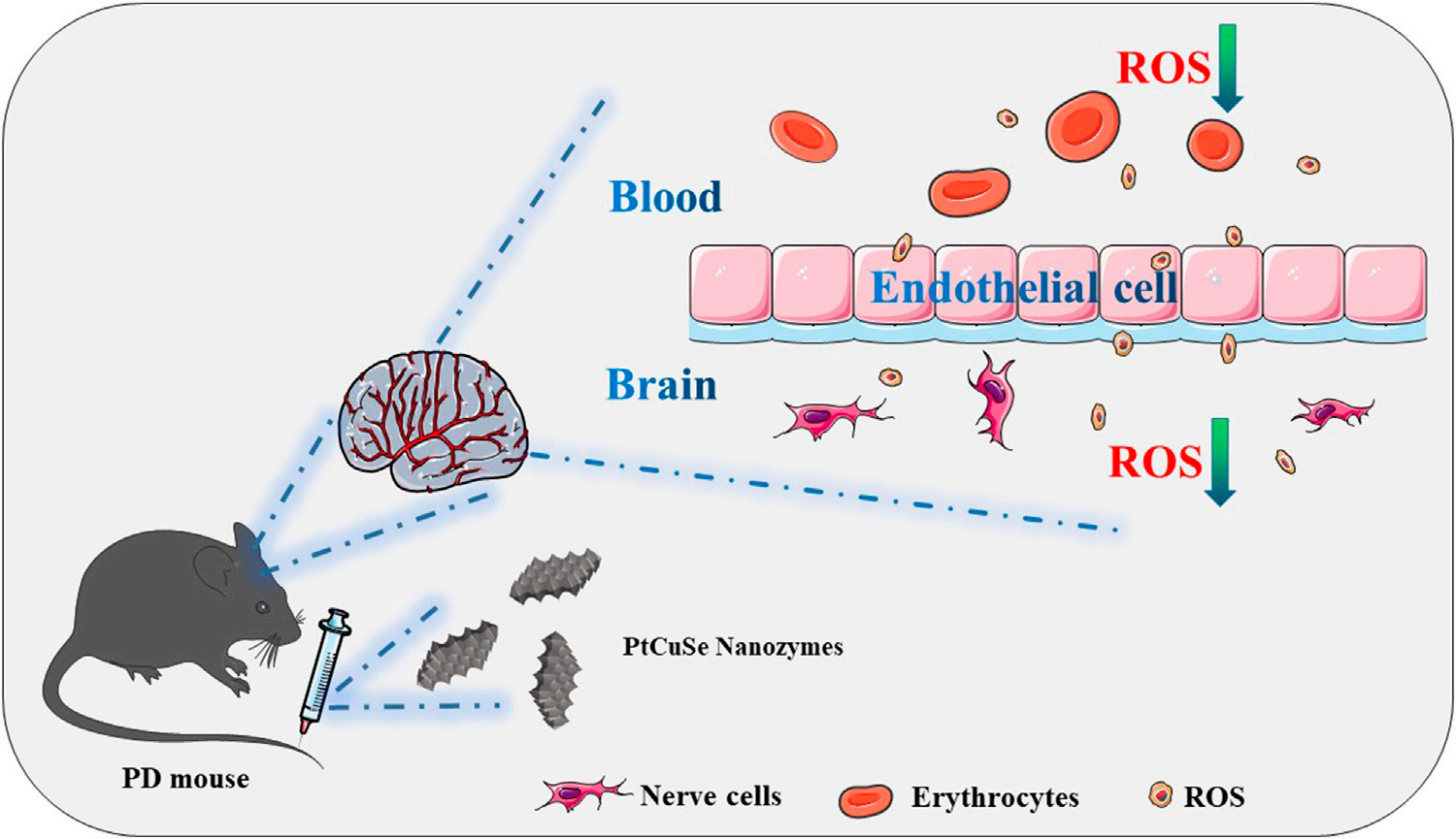
Schematic illustration of the application of the tri-element nanozyme PtCuSe as the cascade catalytic machine for PD symptom relief by ROS depletion.

Herein, the tri-element nanozyme PtCuSe was constructed as an ingenious cascade catalytic machine for the amelioration of Parkinson’s disease-like symptoms. This catalytic machine was employed as the ROS scavenger both in *in vitro* and *in vivo*, effectively relieving oxidative damage and inflammatory reaction of nerve cells and significantly mitigating the behavioral and pathological symptoms of a PD mouse model (Scheme 1).

## 2 Results and discussion

**FIGURE 1:**
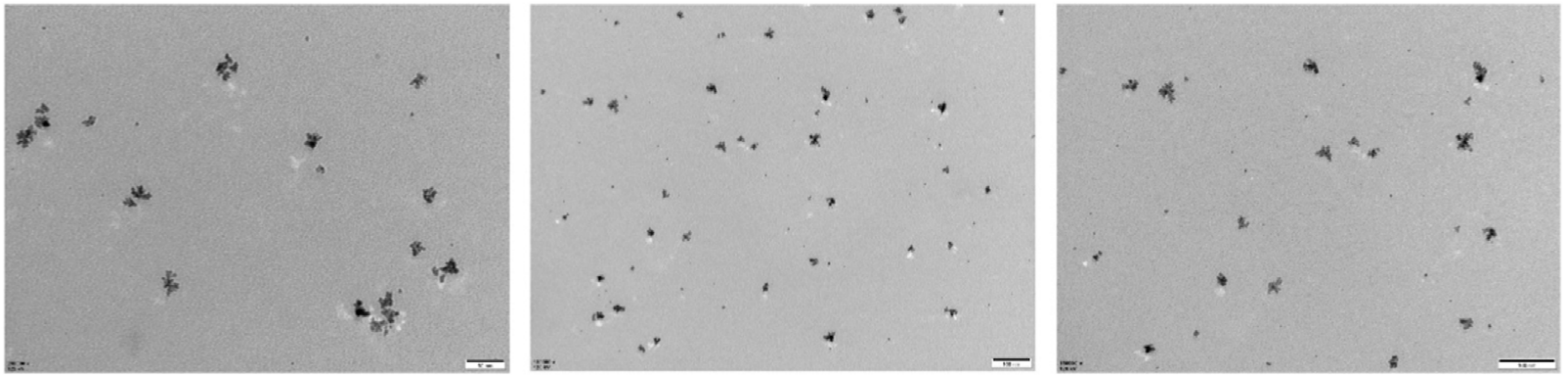
Transmission electron microscope (TEM) images of PtCuSe nanozymes.

**FIGURE 2:**
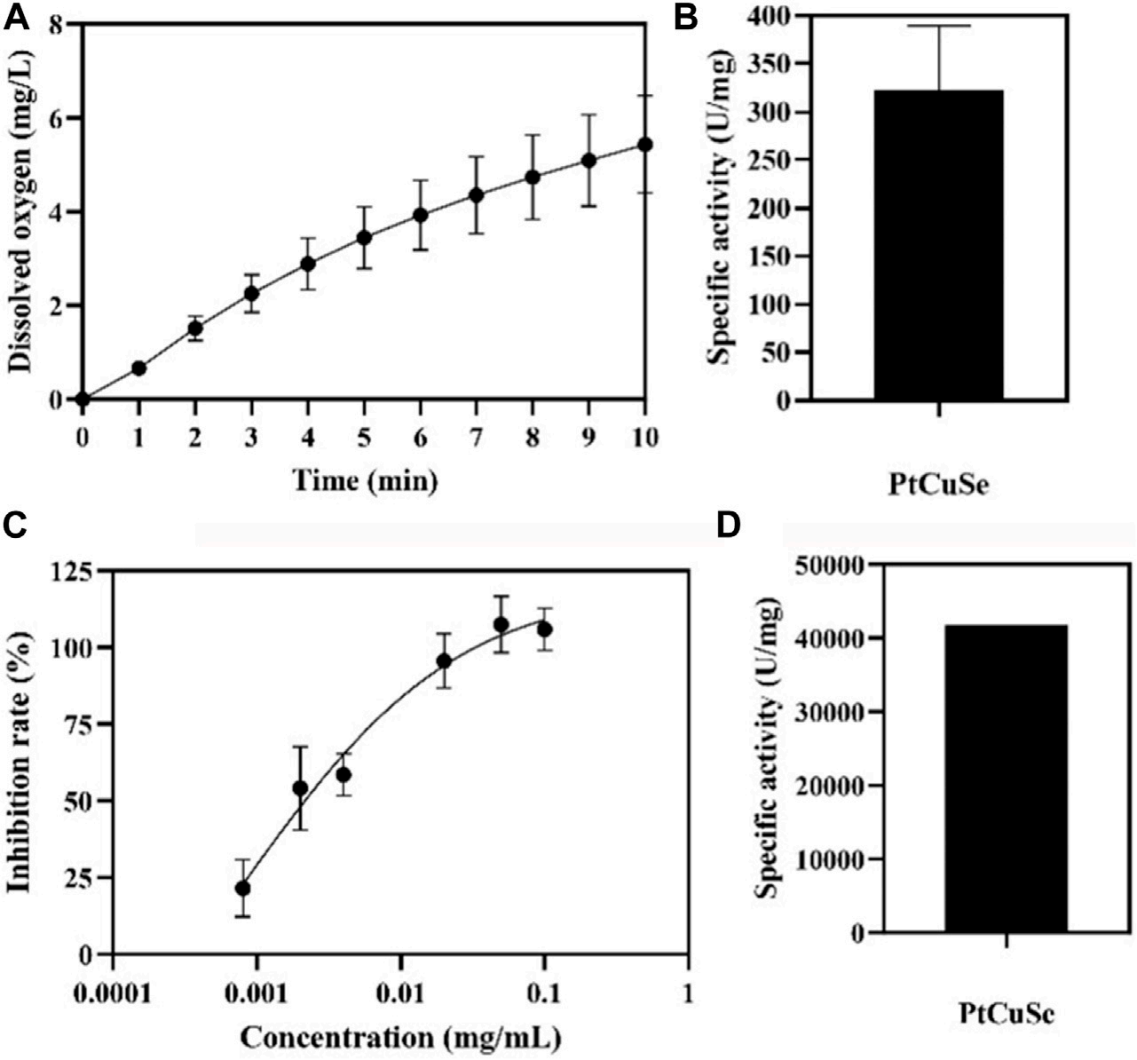
Enzymatic-like activity characterization of PtCuSe nanozyme. **(A,B)** CAT-like activity and **(C,D)** SOD-like activity of PtCuSe nanozyme.

PtCuSe nanozyme is a successful conversion based on PtCu nanoparticles, which not only retains the original advantages of low cytotoxicity but also has higher biological activity. Observation under an electron microscope showed that the structure of PtCuSe is regular and well-dispersed, and its surface character is of obvious consistency (Figure 1). The antioxidant capacity of PtCuSe is reflected in its catalytic activity for hydrogen peroxide reduction (CAT-like), superoxide dismutase (SOD-like), and free radical scavenging. PtCuSe predominantly achieves antioxidant function through the following two aspects: first, it encourages the decomposition of H_2_O_2_, which is a CAT-like activity and well reflected through the detection of the dissolved oxygen level. The dissolved oxygen content demonstrated a positive correlation with time and increased gradually. At the beginning of the recording, the dissolved oxygen content was 0, and when the reaction progresses, the dissolved oxygen approached 6 mg/L at 10 min. This exciting curve confirmed that PtCuSe is stable, strongly efficient, and extremely durable, and even if the concentration is very low, it still reaches a high level of enzyme activity. According to calculations, the catalytic capacity of H_2_O_2_ decomposition into H_2_O and O_2_ per mg of PtCuSe is almost identical to 320 U CAT. This result is amazing because such a high biological enzyme equivalent is considerably beyond expectations, which indicates that PtCuSe has substantial biological activity and broad research value (Figures 2A, B, respectively).

The rate of superoxide anion reduction is strongly associated with the activity of xanthine oxidase which is inhibited by SOD. Therefore, SOD-like activity is normally reflected by detecting the degree of inhibition of xanthine oxidase. The inhibition rate gradually increases with the increase in concentration and reaches 100% when the concentration of PtCuSe is 0.1 mg/mL. According to the inhibition curve, the SOD activity in the sample was calculated, and the activity of PtCuSe nanozyme per milligram was more than 40,000 U SOD. In conclusion, PtCuSe not only obtains an extremely high CAT-like activity but also possesses a potent superoxide dismutase activity, which prevents intracellular oxygen overload and reactive oxygen retention in numerous ways, plays a highly efficient antioxidant role, and blocks the pathological process believed to lead to PD (Figures 2C, D).

**FIGURE 3:**
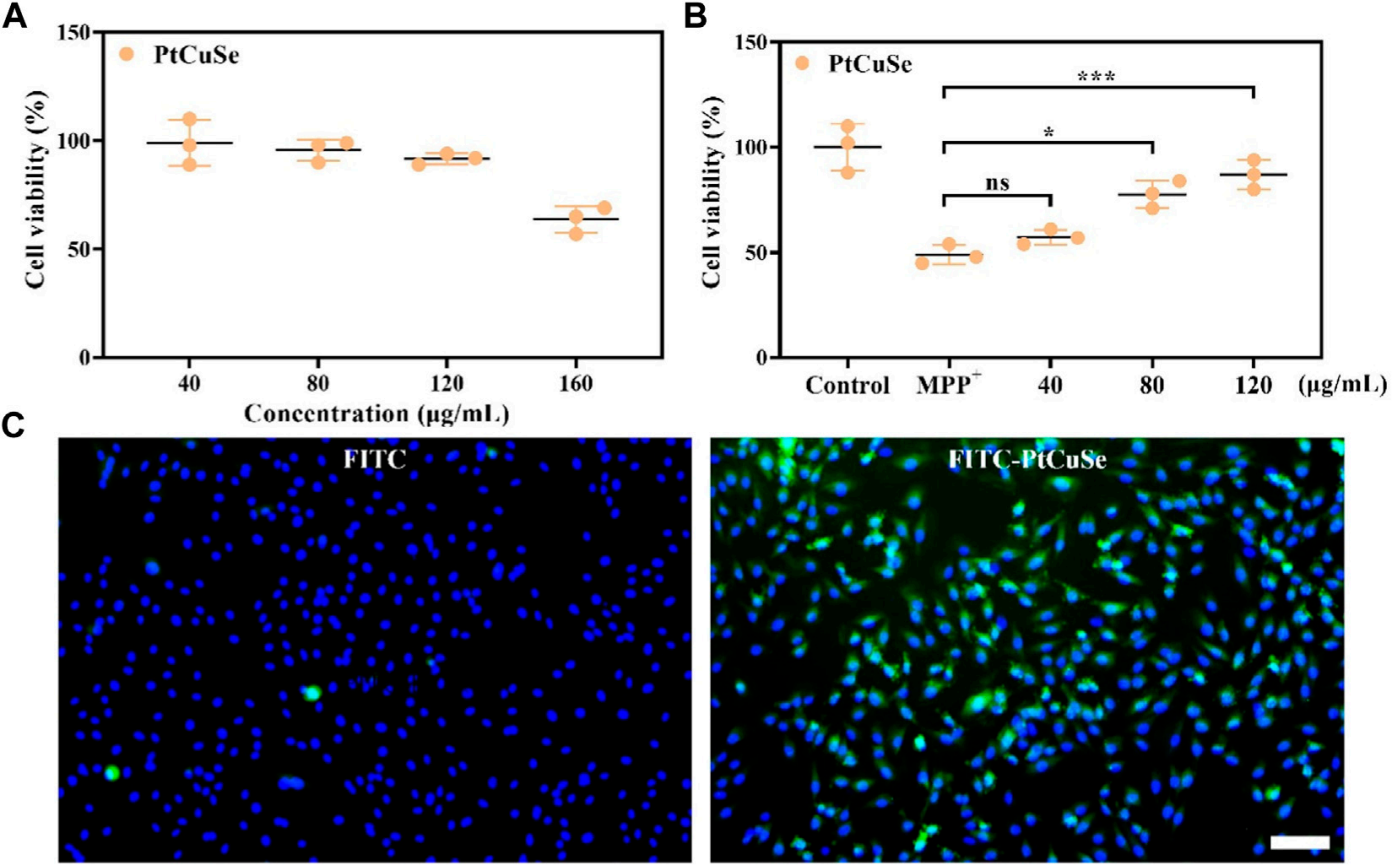
*In vitro* studies showing the neuron protection and cell uptake ability of PtCuSe. **(A)** Cell viability of SH-SY5Y cells under PtCuSe treatments of various concentrations. **(B)** Cell viability of SH-SY5Y cells under different treatment strategies and various concentrations. **(C)** Cell uptake of PtCuSe detected by fluorescent staining. The scale bar is 50 μm.

MTT assay was then employed to detect the cytotoxicity of PtCuSe in *in vitro* culture with different PtCuSe concentration gradients, and the appropriate concentration was determined by cell viability. When the concentration was less than 120 μg/mL, the cytotoxicity was negligible, so we concluded that this was an adequate dosing concentration for experimental requirements (Figure 3A). MTT assay also suggested that the cell viability was significantly reduced after treatment with MPP^+^. However, when we incubated these cells with a range of concentrations of PtCuSe (40–120 μg/mL) in advance, a dose-dependent increase in cell viability could be observed (Figure 3B). Afterward, PtCuSe was again labeled with FITC to form FITC–PtCuSe composites and administered into cultured cells *in vitro*. A bright green fluorescence was observed under the laser confocal microscope, and after adjusting the field of view, it was found with great satisfaction that these fluorescence signals were located in the cell interior. The reason why there was high fluorescence in cells is that the unique nanometer scale of PtCuSe is satisfactory for cell endocytosis, so it is competent to have an extremely high cellular uptake rate, and it is this excellent cellular uptake rate that is the premise of PtCuSe to play an efficient role in the cell (Figure 3C).

**FIGURE 4:**
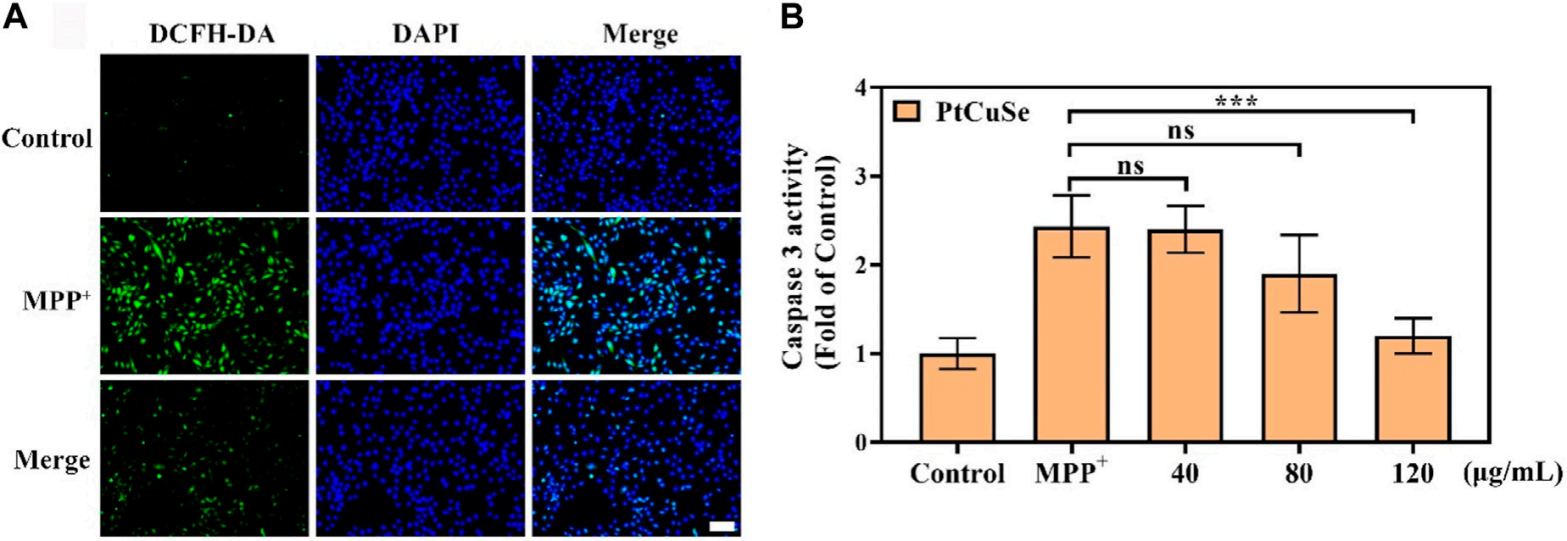
ROS clearance and protective ability against cell apoptosis after treatment with PtCuSe. **(A)** Intracellular ROS levels stained by DCFH-DA and detected using a laser scanning confocal microscope. The scale bar is 50 μm. **(B)** Caspase-3 activity in SH-SY5Y cells after various treatments.

Based on these successful data, we attempt to investigate the scavenging capability of PtCuSe for intracellular ROS. 1-Methyl-4-phenylpyridinium (MPP^+^) was selected as a neurotoxin to generate neuronal cell damage phenotypes, which can lead to the increase in intracellular reactive oxygen species concentration and induce apoptosis of human neuroblastoma SH-SY5Y. A measure of 2 mM MPP^+^ was added to the cell culture medium and co-incubated with the cells. As shown in the figure, the cell death was clearly evident. However, if the cells were treated with PtCuSe in advance and then co-incubated with MPP^+^, the cell survival rate was substantially increased, which verified that PtCuSe had an excellent anti-neurotoxin MPP^+^ effect. Then, 2-7-dichlorodihydrofluorescein diacetic acid (DCFH-DA) was added to monitor the oxidation status of cells to reflect the ROS content and to investigate the role of ROS in cell death induced by neurotoxin MPP^+^ and the protection of cells by PtCuSe. The high content of ROS in MPP^+^-treated SH-SY5Y cells occurred during cell death corresponding to SH-SY5Y incubated with PtCuSe composites. The content of ROS significantly diminished, which was comparable to the result of MPP^+^, and confirmed the capability of PtCuSe composites to clear ROS (Figure 4A). Caspase-3 is an apoptotic protein, which plays an irreplaceable role in the occurrence and regulation of cell apoptosis. Detection of its expression variation is capable of demonstrating the defensive effect of PtCuSe on MPP^+^. As shown in the diagram, caspase-3 expression was significantly increased in SH-SY5Y cells treated with MPP^+^ alone and markedly lower in PtCuSe-treated cells (Figure 4B). In conclusion, MPP^+^ neurotoxin increased the content of intracellular ROS and caspase-3 and induced cell apoptosis, but PtCuSe promoted the decomposition of ROS to decrease intracellular ROS, prevented the expression of caspase-3, and diminished cell apoptosis.

**FIGURE 5:**
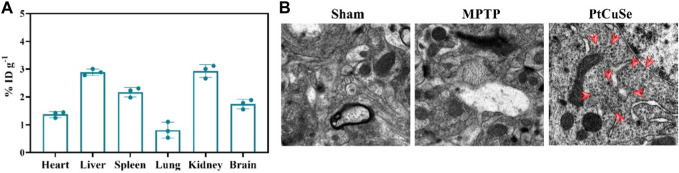
Biological distribution and brain enrichment effects after intravenous injection of PtCuSe nanoparticles. **(A)** Biodistribution of PtCuSe nanoparticles in major organs and brain detected by ICP-MS. **(B)** Bio-TEM images of the ultrathin section of brain lesion areas. The red arrows show the distribution of PtCuSe nanoparticles in the brain.

BBB plays an important role in maintaining the healthy physiological state of the brain. However, BBB also influences or even prohibits the availability of PD therapeutic drugs by the brain. Thus, we then examined the BBB traverse ability of the nanosystem by detecting the biodistribution of PtCuSe in the brain and other organs. PD mouse models were established, and inductively coupled plasma mass spectrometry (ICP-MS) analysis was performed after *i.v.* administration of nanoparticles and obtaining major organs. As shown in Figure 5A, PtCuSe had desirable normalized dosage accumulation. Notably, most of the injected PtCuSe accumulated in the liver and kidney, which might attribute to the renal and hepatic uptake due to its nano-sized hydrophilicity diameter. In addition, bio-TEM also showed that the PtCuSe nanoparticles with high contrast inside the brain, indicating the desirable brain targeting capability of PtCuSe (Figure 5B).

**FIGURE 6:**
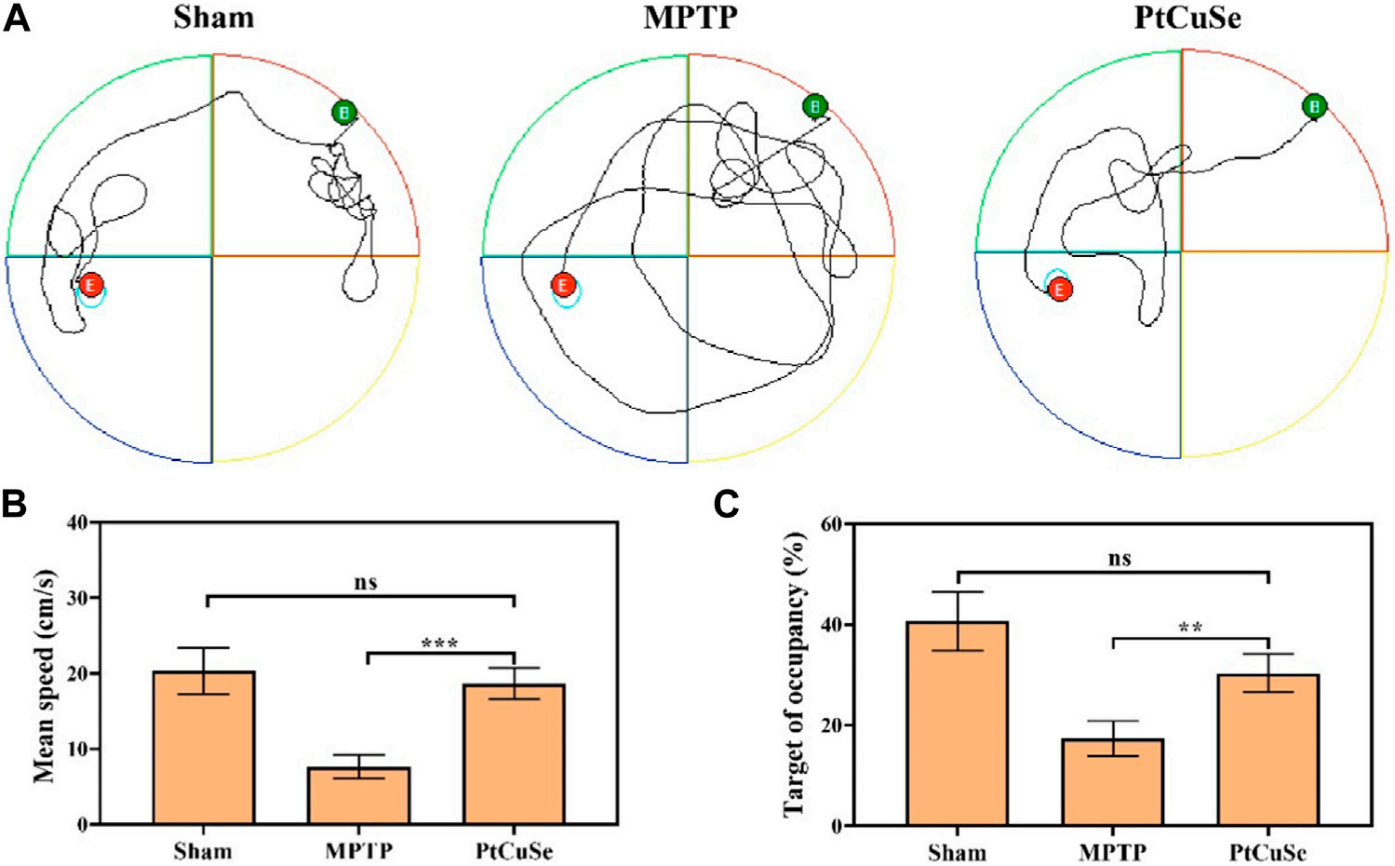
Behavioral assessment of PD mice after treatment with different formulations. The Morris water maze test was used to investigate the athletic and memory ability of PD mice. **(A)** Representative path tracing of mice, **(B)** mean time spent on the target quadrant, **(C)** and the relative time spent on the target quadrant.

Subsequently, the PD mouse model was established by MPTP stimulation, and PD-associated behaviors were assessed by the Morris water maze and open field test after treatment. These animals were divided into three groups: healthy mice (Sham group), MPTP-induced PD mice (MPTP), and PD mice *i.v.* injected with PtCuSe nanozymes (PtCuSe). PD mice exhibited random and disordered motor pathways and could not find the platform timely (Figure 6A). In contrast, the PtCuSe-treated mice reached the platform in a spatially oriented manner. Furthermore, the PtCuSe-treated mice showed improved mean speed (Figure 6B) and target of occupancy (Figure 6C) after treatment, indicating that PtCuSe significantly rescued motor impairments and memory loss in PD mice.

**FIGURE 7:**
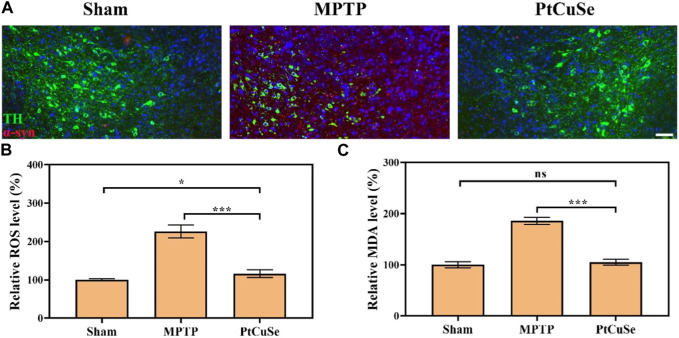
Pathological evaluation of PD mice after treatment with different formulations. **(A)** Co-immunoreactivity analysis after staining the brain sections with an anti-α-syn antibody and anti-TH antibody. The scale bar is 50 μm. **(B)** ROS levels in the SNpc. **(C)** MDA levels in the SNpc region.

To explore the therapeutic efficacy of PtCuSe, the PD mouse model was established via intraperitoneal injection of MPTP. The loss of dopaminergic neurons is the most immediate characteristic of PD, which is reflected by the reduction of TH-positive neurons in SNpc. In addition, the severity of PD can also be reflected by α-syn accumulation as the result of its close relationship with the loss of TH-positive neurons. Therefore, we evaluated the TH and α-syn levels in the SNpc and ST of different groups by co-immunofluorescence. Immunofluorescence in the SNpc suggested that the MPTP-treated mice showed reduced TH+ neurons but elevated α-syn levels, while the contents were reversed after PtCuSe treatment to an extent similar to those of healthy mice (Figure 7A). In addition, ROS and malondialdehyde (MDA) levels were also detected. It suggested that MPTP significantly elevated the peroxidation levels in the lesion site of the brain, which was reversed by the treatment of PtCuSe nanozymes (Figures 7B, C).

## 3 Conclusion

In conclusion, we successfully synthesized PtCuSe nanozymes with SOD- and CAT-like activities, which can be applied as an excellent cascade catalytic machine for the depletion of ROS in the lesion site of PD. The PtCuSe nanozyme has efficient cellular uptake in neurons, consistently and stably removing intracellular ROS and substantially enhancing cell viability. In addition, *in vivo* studies suggest that the PtCuSe nanozyme has satisfactory brain enrichment, thus alleviating both behavioral and pathological symptoms in PD mice after intravenous administration. Therefore, it is believed that PtCuSe provides a novel approach to the treatment and research of PD and opens up avenues for the application of a three-element nanozyme in the treatment of PD and other neurodegenerative diseases. In the future, PtCuSe is expected to be used in the treatment of early Parkinson’s disease by injection to achieve the therapeutic goal of early treatment, early prevention, and early rehabilitation.

## 4 Materials and methods

### 4.1 Materials

DMEM and FBS were purchased from Gibco. Thiazolyl blue tetrazolium bromide (MTT) was purchased from Sigma-Aldrich. SH-SY5Y cells were purchased from Wuhan Pricella Life Technology Co., Ltd. 1-Methyl-4-phenyl-1,2,3,6-tetrahydropyridine (MPTP) hydrochloride and 2′,7’-dichlorodihydrofluorescein diacetate (DCFH-DA) were purchased from Sigma-Aldrich.

### 4.2 ROS detection

SH-SY5Y cells were treated with drugs at optimized concentrations for 6 h, followed by incubation with MPP^+^ for another 4 h, and then stained with 3 μM DCFH-DA for 60 min at 37°C. Afterward, the ROS content in the cells was evaluated using a confocal laser scanning microscope (FV1200, Olympus, Japan).

### 4.3 Cell viability assay

SH-SY5Y cells were seeded into 96-well plates at a density of 8,000 cells/well overnight. Drugs were added to the cells for 6 h, and then, 2 mM MPP^+^ was subsequently added and incubated for another 24 h. A measure of 20 μL of MTT solution (5 mg/mL) was added into each well and incubated at 37°C for another 4 h. Finally, dimethyl sulfoxide (DMSO) was used to dissolve the formed formazan crystal, and the absorbance at 492 nm was measured using a GF-M3000 microplate reader (Caihong, Shandong, China).

### 4.4 Construction and treatment of the Parkinson’s disease (PD) model

All animal experiments were approved by the Institution Animal Ethics Committee of Zhengzhou University. Six week old C57BL/6 mice (Sipeifu Biotechnology Co., Ltd., Beijing, China) were fed in cages with controlled temperature and humidity. Before the investigation, the mice were subjected to rotarod performance on the rotation rod, and the mice which exhibited behavioral consistency were selected for subsequent studies. The mice were subjected to intraperitoneal MPTP injection (35 mg/kg/day for 5 consecutive days) to induce a PD-like phenotype.

### 4.5 Brain tissue distribution

PD-like phenotype mice were injected with PtCuSe (8 mg/kg) through the tail vein. Afterward, the mice in each group were euthanized, and the main organs were collected 24 h after injection. Then, the tissues were weighed and homogenized to calculate the percentage of injected dose per gram of tissue (%ID g^−1^) by ICP-MS (Agilent 7800, China). Three mice from each group were euthanized for brain tissue collection.

### 4.6 Immunofluorescence analysis

The brains of the treated mice were collected and fixed with 4% paraformaldehyde for 72 h. Then, the SNpc tissues were embedded, and paraffin sections of 10 μm thickness were obtained. The block process was performed after antigen retrieval, in which the slides were incubated in 10% rabbit serum for 30 min at room temperature. Then, primary antibodies for TH (GB12181, Servicebio, China) and α-syn (ab212184, Abcam, China) were applied, and then the slices were washed and incubated with secondary antibodies. After counterstaining with DAPI for 10 min and sealing, the slides were observed using a panoramic section scanner.

### 4.7 Statistical analysis

GraphPad Prism 8.0.2 was utilized for all statistical analyses. The outcomes were compared via Student’s *t*-tests. The significance was measured as *p < 0.01, **p < 0.005, ***p < 0.001, and ****p < 0.0001.

## Data Availability

The original contributions presented in the study are included in the article/Supplementary Material; further inquiries can be directed to the corresponding authors.

## References

[B1] Bengoa-Vergniory N. Faggiani E. Ramos-Gonzalez P. Kirkiz E. Connor-Robson N. Brown L. V. (2020). CLR01 protects dopaminergic neurons *in vitro* and in mouse models of Parkinson's disease. Nat. Commun. 11 (1), 4885. 10.1038/s41467-020-18689-x 32985503 PMC7522721

[B2] Chen J. Zhang S. Chen X. Wang L. Yang W. (2022). A self-assembled fmoc-diphenylalanine hydrogel-encapsulated Pt nanozyme as oxidase- and peroxidase-like breaking pH limitation for potential antimicrobial application. Chem 28 (26), e202104247. 10.1002/chem.202104247 35191569

[B3] Cheng G. Liu Y. Ma R. Cheng G. Guan Y. Chen X. (2022). Anti-parkinsonian therapy: Strategies for crossing the blood-brain barrier and nano-biological effects of nanomaterials. Nanomicro Lett. 14 (1), 105. 10.1007/s40820-022-00847-z 35426525 PMC9012800

[B4] Dilliard S. A. Siegwart D. J. (2023). Passive, active and endogenous organ-targeted lipid and polymer nanoparticles for delivery of genetic drugs. Nat. Rev. Mater 8, 282–300. 10.1038/s41578-022-00529-7 36691401 PMC9850348

[B5] Ding S. Liu Z. Huang C. Zeng N. Jiang W. Li Q. (2021). Novel engineered bacterium/black phosphorus quantum dot hybrid system for hypoxic tumor targeting and efficient photodynamic therapy. ACS Appl. Mater Interfaces 13 (8), 10564–10573. 10.1021/acsami.0c20254 33605723

[B6] Dringen R. Brandmann M. Hohnholt M. Blumrich E. (2015). Glutathione-dependent detoxification processes in astrocytes. Neurochem. Res. 40, 2570–2582. 10.1007/s11064-014-1481-1 25428182

[B7] Emin D. Zhang Y. P. Lobanova E. Miller A. Li X. Xia Z. (2022). Small soluble α-synuclein aggregates are the toxic species in Parkinson’s disease. Nat. Commun. 13 (1), 5512. 10.1038/s41467-022-33252-6 36127374 PMC9489799

[B8] Gao X. Liu Y. Li Y. Jin B. Jiang P. Chen X. (2023). Piezoelectric nanozyme for dual-driven catalytic eradication of bacterial biofilms. ACS Appl. Mater Interfaces 15 (11), 14690–14703. 10.1021/acsami.2c21901 36880988

[B9] Han G. Bai K. Yang X. Sun C. Ji Y. Zhou J. (2022). “Drug‐Carrier” synergy therapy for amyloid‐ *β* clearance and inhibition of tau phosphorylation via biomimetic lipid nanocomposite assembly. Adv. Sci. (Weinh) 9 (14), e2106072. 10.1002/advs.202106072 35307993 PMC9108666

[B10] He C. Lin X. Mei Y. Luo Y. Yang M. Kuang Y. (2022). Recent advances in carbon dots for *in vitro*/vivo fluorescent bioimaging: A mini-review. Front. Chem. 10, 905475. 10.3389/fchem.2022.905475 35601546 PMC9117726

[B11] He X. Wang X. Yang L. Yang Z. Yu W. Wang Y. (2022). Intelligent lesion blood-brain barrier targeting nano-missiles for Alzheimer's disease treatment by anti-neuroinflammation and neuroprotection. Acta Pharm. Sin. B 12 (4), 1987–1999. 10.1016/j.apsb.2022.02.001 35847512 PMC9279705

[B12] Hu Y. Wang K. Ye C. (2022). Four-in-One" nanozyme and natural enzyme symbiotic system of Cu(2-x) Se-GOx for cervical cancer therapy. Chem 28 (1), e202102885. 10.1002/chem.202102885 34773414

[B13] Huang Y. Liu C. Pu F. Liu Z. Ren J. Qu X. (2017). A GO-Se nanocomposite as an antioxidant nanozyme for cytoprotection. Chem. Commun. (Camb) 53 (21), 3082–3085. 10.1039/c7cc00045f 28243649

[B14] Li Q. Liu Y. Dai X. Jiang W. Zhao H. (2021). Nanozymes regulate redox homeostasis in ROS-related inflammation. Front. Chem. 9, 740607. 10.3389/fchem.2021.740607 34746091 PMC8567209

[B15] Li Q. Shao X. Dai X. Guo Q. Yuan B. Liu Y. (2022). Recent trends in the development of hydrogel therapeutics for the treatment of central nervous system disorders. NPG Asia Mater 14 (1), 14. 10.1038/s41427-022-00362-y

[B16] Li Q. Wu T. Fan X. Guo X. Jiang W. Fan K. (2022). Multifaceted nanozymes for synergistic antitumor therapy: A review. Mater. Des. 224, 111430. 10.1016/j.matdes.2022.111430

[B17] Liu Y.-Q. Mao Y. Xu E. Jia H. Zhang S. Dawson V. L. (2021). Nanozyme scavenging ROS for prevention of pathologic α-synuclein transmission in Parkinson’s disease. Nano Today 36, 101027. 10.1016/j.nantod.2020.101027

[B18] Niu B. Liao K. Zhou Y. Wen T. Quan G. Pan X. (2021). Application of glutathione depletion in cancer therapy: Enhanced ROS-based therapy, ferroptosis, and chemotherapy. Biomater 277, 121110. 10.1016/j.biomaterials.2021.121110 34482088

[B19] Olson K. E. Namminga K. L. Lu Y. Thurston M. J. Schwab A. D. de Picciotto S. (2021). Granulocyte-macrophage colony-stimulating factor mRNA and Neuroprotective Immunity in Parkinson's disease. Biomater 272, 120786. 10.1016/j.biomaterials.2021.120786 PMC8382980 33839625

[B20] Peter C. Braidy N. Zarka M. Welch J. Bridge W. (2015). Therapeutic approaches to modulating glutathione levels as a pharmacological strategy in Alzheimer's disease. Curr. Alzheimer Res. 12, 298–313. 10.2174/1567205012666150302160308 25731620

[B21] Sun H. Wang Y. Hao T. Wang C. Wang Q. Jiang X. (2016). Efficient GSH delivery using PAMAM-GSH into MPP-induced PC12 cellular model for Parkinson's disease. Regen. Biomater. 3, 299–307. 10.1093/rb/rbw032 27699060 PMC5043156

[B22] Wu J. Cui X. Ke P. C. Mortimer M. Wang X. Bao L. (2021). Nanomaterials as novel agents for amelioration of Parkinson’s disease. Nano Today 41, 101328. 10.1016/j.nantod.2021.101328

[B23] Xiong S. Liu W. Zhou Y. Mo Y. Liu Y. Chen X. (2020). Enhancement of oral bioavailability and anti-Parkinsonian efficacy of resveratrol through a nanocrystal formulation. Asian J. Pharm. Sci. 15 (4), 518–528. 10.1016/j.ajps.2019.04.003 32952674 PMC7486547

[B24] Xue Z. Y. Yu J. L. Xia Q. Q. Zhu Y. Q. Wu M. X. Liu X. (2022). Color-tunable binary copolymers manipulated by intramolecular aggregation and hydrogen bonding. ACS Appl. Mater Interface 14, 53359–53369. 10.1021/acsami.2c17600 36383092

[B25] Yang Q. Y. Wan C. Q. Wang Y. X. Shen X. F. Pang Y. H. (2023). Bismuth-based metal-organic framework peroxidase-mimic nanozyme: Preparation and mechanism for colorimetric-converted ultra-trace electrochemical sensing of chromium ion. J. Hazard Mater 451, 131148. 10.1016/j.jhazmat.2023.131148 36889075

[B26] Yu J. L. Wu M. X. Xue Z. Y. Xia Q. Q. Liu X. Wang X. H. (2022). Supramolecular assembly‐induced emission enhancement vesicles regulated by pincer‐like hosts containing pillar[5]arenes. Adv. Opt. Mater 10, 2201496. 10.1002/adom.202201496

[B27] Yuan J. Liu H. Zhang H. Wang T. Zheng Q. Li Z. (2022). Controlled activation of TRPV1 channels on microglia to boost their autophagy for clearance of alpha-Synuclein and enhance therapy of Parkinson's disease. Adv. Mater 34 (11), e2108435. 10.1002/adma.202108435 35023596

[B28] Zhang Y. Li Q. Han C. Geng F. Zhang S. Qu Y. (2022). Superoxide dismutase@zeolite imidazolate framework-8 attenuates noise-induced hearing loss in rats. Front. Pharmacol. 13, 885113. 10.3389/fphar.2022.885113 35662706 PMC9159373

[B29] Zhu Y. Wang Z. Zhao R. Zhou Y. Feng L. Gai S. (2022). Pt decorated Ti_3_C_2_T_x_ MXene with NIR-II light amplified nanozyme catalytic activity for efficient phototheranostics. ACS Nano 16 (2), 3105–3118. 10.1021/acsnano.1c10732 35040328

